# The Challenge to Translate OMICS Data to Whole Plant Physiology: The Context Matters

**DOI:** 10.3389/fpls.2017.02146

**Published:** 2017-12-13

**Authors:** Marcelo N. do Amaral, Gustavo M. Souza

**Affiliations:** Department of Botany, Institute of Biology, Federal University of Pelotas, Pelotas, Brazil

**Keywords:** downward causation, emergent properties, hierarchical systems, plant signaling, systems biology

## Introduction: some challenges

The exponential development of high-throughput technologies in the last decades, supporting and improving the OMICS science, has allowed uncovering successfully the complexity of the organizational network patterns in the cell's metabolism to the plant phenome, founding the science of system biology (Mochida and Shinozaki, 2011). Further, the huge data sets and growing computational power have stimulated scientists to glimpse about how plants respond to the environmental changes, and how such knowledge could engender new technologies, for instance, to increase crop yields (Edwards and Batley, 2004; Tardieu et al., 2017). Through these technologies, researchers are describing deeply the different hierarchical levels of plant organization, improving the possibility to predict the behavior of whole plant (phenome). Based on extensive analyses of gene expression (genome and transcriptome) and/or metabolic networks (metabolome), it has been possible to monitor and control cellular responses to genetic perturbations or environmental changes (Fukushima et al., 2009).

**Figure 1:**
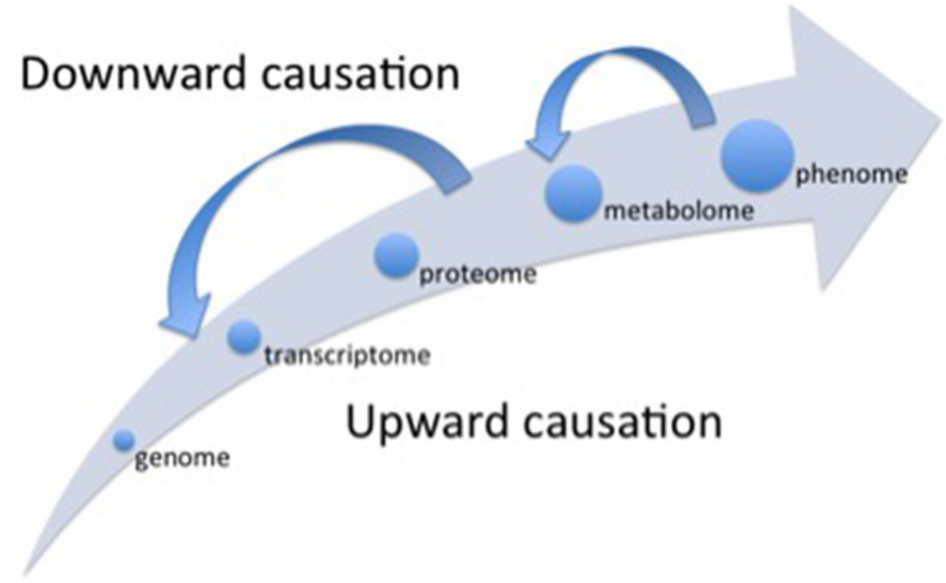
Representation of the different levels of organization (hierarchical scales) considered in different OMICS scales. Alongside the main arrow, the different scales follow an upward causation chain, when the lower level (e.g., genome) “determines” the subsequent higher-level properties (e.g., transcriptome). The small arrows depicted in the opposite direction of the main arrow represent the top-down effects (downward causation) regulating the processes operating at lower scales of organization. The whole picture represents the concept of “non-privileged level of causation” (Noble, 2008).

However, different constrains can make both the predictability and the controllability difficult from the bottom-up cause-effect approach that underpins the deterministic view of science based on an upward chain of causality (Figure 1) (Noble, 2008; Sheth and Thaker, 2014). The first “bottleneck” is how to integrate the massive datasets from molecular high-throughput technologies with the growing high-throughput information on the crop scale, i.e., plant phenomics (Fukushima et al., 2009; Tardieu et al., 2017), which is a typical problem of finding a proper (if it does exists indeed) scaling law (Souza et al., 2016).

The second constrain comes from the common assumption in biochemical models that the system sampled should be in a metabolic steady-state at a given moment, characterized by constant metabolite levels, and that the different metabolic pathways operate in isolation (Toubiana et al., 2013), which is an obvious oversimplification. For instance, at intracellular level, compartmentalization into organelles enables differences in metabolite concentrations acting as a barrier to passive diffusion between organelles and cytoplasm, creating a non-homogenous cellular metabolic space (Sweetlove and Fernie, 2013; de Souza et al., 2017). Moreover, each metabolic pathway, somehow, is integrated in a dynamical metabolic network (Toubiana et al., 2013), which is challenging for static networks mathematical models that often bypass the network modulation over time. For instance, the stomatal movement depends on a range of environmental and endogenous plant stimuli that affect the internal networks at multiple levels of cellular spatio-temporal organization, generating species-specific responses to combined external stimuli (Merilo et al., 2014). From the modeling of a single guard cell at steady-state, researches seek for elucidate how these interactions determine the phenotype of plants. However, due of this hierarchy of scales, the interpretation of a large set of data from OMICS tools becomes quite difficult, then it is necessary to develop new methods to allow investigations of dynamic aspects of large scale models (Medeiros et al., 2015).

The first “bottleneck” refers to the problem of emergent properties at the higher level organizations of the system that are not fully determined by the properties of the lower levels (Souza et al., 2016), for example, changes in the transcriptome or in the proteome do not always result in respective alterations in the metabolome (biochemical phenotype) that exhibits its own dynamics (Ryan and Robards, 2006). Additionally, there is the influence of downward causation processes (Noble, 2008) (Figure 1), when higher levels of organization affect the functioning of the lower levels. For instance, interlocked transcriptional/translational feedback loops are involved in the generation of circadian rhythm in plants, and the functional clock (higher level) controls a wide range of cellular processes such as gene expression (lower level; Fukushima et al., 2009).

The second constraint is related to the different sources of “uncertainty” operating in different levels of plant organization, blurring the predictability from lower levels. For instance, at cellular level, the sources of uncertainty emerge from the spontaneous thermodynamical noise of molecular activity constraining the flux of distributions in metabolic networks (Hoppe et al., 2007), interactions between genes that enable alternative routes for the same phenotype (Kohl et al., 2010), and epigenetic effects changing genes expression (Crisp et al., 2016). Moreover, at the level of whole plant integration, there are many types of long-distance signaling processes (chemical and electrical) over toping each other and engendering a highly complex informational network that feedback on the regulation of cells metabolism (Choi et al., 2016).

Further, it's worth to consider that, especially under stressful conditions, the different sources of external “noise” often affect the way that plants respond to environmental changes (Bertolli and Souza, 2013; Prasch and Sonnewald, 2015). Environmental fluctuations potentiate the accumulation of conserved cellular signals such as reactive oxygen species (ROS) and the modulation of intracellular Ca^2+^ (Chi et al., 2015; de Souza et al., 2017). Different types of ROS and oxidized molecules produced in different subcellular compartments, together with a spatial and temporal modulation of Ca^2+^ elicit different transcriptional responses and, in several cases, the expression of nuclear genes can be altered without altering the total concentration of the signaling molecule in the cell as a whole (Tuteja and Mahajan, 2007; Leister, 2012).

## Intercellular and whole plant signaling potentiate the challenges to integrate OMICS information across scales

In addition to intracellular complexity, the interaction between neighboring cells plays an important role in the responses to environmental conditions, and the plant metabolism organization as a whole. In plants, plasmodesmata connect the cytoplasts of adjacent cells across the cell wall, allowing intercellular transport and communication to adjacent cells within a tissue or organ, allowing exchange of small molecules, such as ions, sugars, and phytohormones, as well as larger molecules, including proteins, RNA, and viruses (Brunkard et al., 2013). The exchanges of different types of molecules among cells generate, within the same tissue or organ, different gradients of molecules and metabolites, increasing the complexity of physiological processes. In addition, the same type of signal often induces different calcium-dependent responses between two cells of the same type (Gilroy and Trewavas, 2001). Intercellular communication through plasmodesmata plays a crucial role in specifying the fate of cells, as well as in different responses of the same tissue to environmental conditions (Pyott and Molnar, 2015). An example of this can be seen in one of the mechanisms of root development regulation through the short-range cell-to-cell movement of *miR165/6* (Carlsbecker et al., 2010). The expression of mobile *miR165/6* in the endoderm results in a morphogenic gradient, which extends into the xylem layers toward the root center. This generates an opposite *PHABULOSA* (PHB) expression gradient (regulated by *miR165/6*), which therefore has a higher concentration in internal xylem tissue. Thus, xylem tissue within the stele is defined, among other factors, by the expression of *PHB*, which is restricted to xylem and procambium by *miR165/6*, specifically expressed in the endoderm.

This intercellular communication through non-autonomous mobile signals adds a further challenge to OMICS approaches, because organs and plant tissues present a great heterogeneity in expression patterns and metabolite profiles, and this information can be lost upon tissue homogenization for downstream analyses.

Besides local communication mechanisms, plants developed long distance signaling processes that enable communication and systemic responses. This type of communication responds to a wide range of environmental stimuli in which the perceived signals are transmitted to the distal organs, inducing systemic responses. Several messengers have been proposed to mediate this systemic communication in plants such as ROS, electrical signals and Ca^2+^, appearing to be integrated, demonstrating a fast, complex, and finely tuned communication system (Gilroy et al., 2014).

The systemic responses increase system complexity (plant as a whole), and thus increase the uncertainties of the bottom-up predictability models, since physiological changes in specific tissues may have non-local causes. For instance, a local application of high light results in the activation of a ROS wave, allowing an increase in stress tolerance accompanied by the accumulation of photorespiratory amino acids, including Glycine and Serine, in non-stimulated tissues (Suzuki et al., 2013). Examples also demonstrate that Ca^2+^ propagation increase in aerial parts of the plant induced by local root treatment with NaCl, showing propagation kinetics differences of leaf-to-leaf (Xiong et al., 2014). The miRNAs also act on systemic responses, such as *miR399* that function as a signaling molecule between the aerial tissues and roots to regulate the uptake of inorganic phosphates (Pi) (Chiou et al., 2006).

Actually, the cell is the result of the properties that emerge from the complex interactions and spatial structures among the thousands of molecules and enzymes of which it is composed. In addition, the environmental context, whether from outside or inside the plant, contributes to shape the way that information is processed by each cell (Gilroy and Trewavas, 2001), and these properties expand at different scales within the plant (Souza et al., 2016). According to Vítolo et al. (2012), the observation of different scales of plant organization, under the same circumstances, can show remarkable differences in the responses to the same stimuli, allowing different interpretations if considering each scale as isolated (Stressed or not stressed? It's the question…). For instance, when plants of soybean were subjected to drought, it was observed, in one hand, significant decreases in gas exchanges (reflecting reduction in plant growth) but, on the other hand, non-significant alterations in chlorophyll florescence neither in enzymatic antioxidant activity (Bertolli et al., 2014). Therefore, different scales of organization can show different homeostatic capacities when disturbed, supporting the hypothesis that there is not a privileged level of causation in biological systems (Noble, 2012).

## Concluding remarks

The Cartesian method proposed by René Descartes in the seventeenth century set that the first step in order to understand some natural phenomenon is to analyze it, i.e., to decompose the phenomenon in its constitutive parts and to understand them separately. This first step is based on the mechanistic assumption that the ultimate components of a particular phenomenon “determine” the properties of the phenomenon itself, supporting the raising of the reductionist approach. The second main step in the Cartesian method is the synthesis, i.e., from knowledge gathered of each isolated part build the “whole picture”. Thus, starting from the Galilean scientific revolution (sixteenth century) until the end of twenty and beginning of twenty-first centuries, occidental science was successful to uncover the layers of complexity underlying to the biological organisms, opening the OMICS era with the genome. But the “whole picture” was not clear yet. As exemplified in the previous sections, some problems have challenged the determinism from below, and then the Cartesian synthesis was boosted to explore higher levels of organization, inaugurating the System Biology thinking. The knowledge that has being built on transcriptome, proteome and, specially, the metabolome (Ryan and Robards, 2006) has showed that the higher levels of organization contribute to regulate the lower levels in a downward causation chain (Figure 1), indicating that there is no a privileged level of causation in the organization of biological systems (Noble, 2012).

Therefore, the main message herein is: the context matters. Whatever the scale of observation is taken (from genes to the whole plant), the interpretation of the data shall consider the context in which the particular scale is embedded. Ultimately, in studies that intent to contribute for improvement of crop yield, the plant phenotype (biomass, root, and shoot architecture and/or the crop yield) should have the final word of the meaning of the changes from the lower levels of organization, since one genotype can be translated in many phenotypes when developed under different environmental conditions (Tardieu et al., 2017). Thus, studies tacking into account specific lower levels of organization should maintain their interpretation restricted to those particular levels, avoiding excessive speculative inferences on the higher levels.

## Author contributions

MdA and GS contributed equally to the discussion of the topic.

### Conflict of interest statement

The authors declare that the research was conducted in the absence of any commercial or financial relationships that could be construed as a potential conflict of interest.
